# Molecular Characteristics of the Serological Weak D Phenotype in Koreans

**DOI:** 10.3390/diagnostics11060920

**Published:** 2021-05-21

**Authors:** Dajeong Jeong, Sujin Oh, Eun Young Song, Yun Ji Hong, Kyoung Un Park

**Affiliations:** 1 Department of Laboratory Medicine, Seoul National University College of Medicine, Seoul 03080, Korea; jdjdj0202@gmail.com (D.J.); sujin5976@naver.com (S.O.); eysong1@snu.ac.kr (E.Y.S.); 2 Department of Laboratory Medicine, Seoul National University Bundang Hospital, Seongnam 13620, Korea

**Keywords:** weak D, partial D, RhD variant, PCR-SSP, real-time PCR

## Abstract

Serological weak D is a reaction of 2+ or less to anti-D reagent and includes weak D and partial D phenotypes. Although identifying the RhD subtype is important for transfusion safety, serological tests are insufficient for defining the RhD subtype, and molecular tests are needed. To analyze the molecular characteristics of D variants in Koreans to facilitate the formulation of individualized transfusion strategies, molecular tests such as RhD genotyping using real-time polymerase chain reaction (PCR) and partial-D and/or weak-D sequence-specific amplification (SSP) were performed on 105 Korean Rare Blood Program (KRBP) patients exhibiting serological weak D. In total, 58 out of 68 serologically determined weak D KRBP patients were typed as having weak D or partial D phenotypes via RhD genotyping. In detail, eight (13.8%) were typed as partial DVa or DBS, nine (15.5%) as weak D type 15, and four others (6.8%) as partial DVI, partial DVII, weak D type 2, or weak D type 41 or 45, whereas the rest (n = 37, 63.8%) was typed as having either weak D or partial D. This suggests that serological weak D Koreans who require transfusion should be treated as D-negative.

## 1. Introduction

Serological weak D is characterized by a negative reaction with anti-D reagent, a reaction of 2+ or less at the initial work-up for RhD serotyping, and a positive reaction at the anti-human globulin (AHG) phase [1,2]. It includes molecularly defined weak D and some partial D.

Identifying the RhD subtype is of great importance due to the immunogenicity of the D antigen, one of the strongest among the blood groups. Weak D is a quantitative defect of RhD antigen expression on the red blood cell (RBC) membrane, while partial D is a qualitative defect of RhD antigen attributed to Rh protein deletions [3]. Weak D types 1, 2, 3, and 4.1 can be safely managed as RhD-positive, whereas weak D types 4.2, 11, and 15 have been reported to produce allo anti-D [4,5]. Meanwhile, the partial D type, which lacks some D epitopes, can induce alloimmunization upon exposure to RhD-positive RBCs [3].

Serological testing is a useful, but not definitive, method for identifying RhD variant subtypes. The test has variable degrees of reactivity to anti-D reagents and cannot accurately distinguish between weak D and partial D. Indeed, the same RhD variant can produce different serological patterns [6]. These inherent limitations of serological techniques necessitate molecular tests for differentiating RhD variant subtypes.

The subtypes of serological weak D have been molecularly characterized and reported to differ considerably by race and ethnicity [7]. For instance, 95% of Caucasians in central Europe with the serological weak D phenotype were classified as weak D type 1, 2, or 3 [3]. Consequently, most serological weak D Caucasians would likely be treated as RhD-positive. By contrast, in Africans, several RhD variants, such as DAU-3 or DAR, are reportedly prevalent, which reflects the heterogeneity of distribution according to subethnicity and geographic region [8,9]. The dominant types of serological weak D in the Chinese population are partial DVI and weak D type 15 [10]. These racial and ethnic differences in RhD variant subtypes may necessitate the establishment of individualized transfusion strategies.

The sporadic weak D types of a few patients in South Korea have been published, i.e., types 15, 17, 33, and 43 as well as an *RHD* variant (NM_016124.3: c.1056C>G). Weak D type 17 was identified in two serologically RhD-negative cases, while type 33, 43, and *RHD* c.1056C>G were serologically weak D. Production of anti-D antibodies in the types 17, 33, 43 and *RHD* c.1056C>G has not been reported yet [11,12,13]. Herein, we investigated the prevalence of serological weak D in Korean patients and analyzed the molecular characteristics of this phenotype using real-time PCR and PCR-SSP to enable the development of individualized transfusion strategies.

## 2. Materials and Methods

### 2.1. Patients

**Figure 1:**
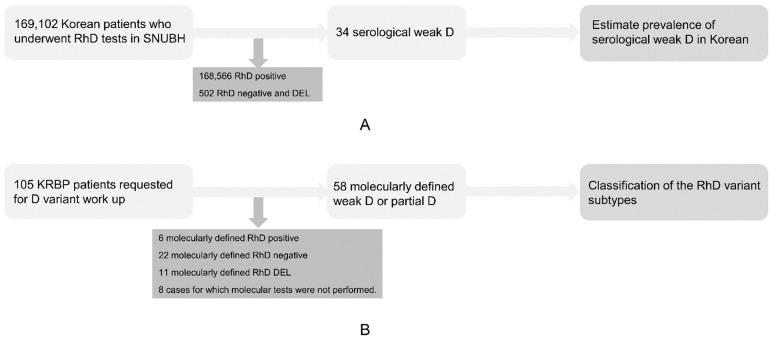
Retrospective study design. (**A**) Among the 169,102 patients who underwent RhD tests in Seoul National University Bundang Hospital from 2013 to 2018, those with RhD-positive and serologically RhD-negative phenotypes, which potentially includes DEL, were excluded. Consequently, 34 of the 169,102 patients were typed as having serologically weak D. (**B**) A total of 105 patients were referred for D variant work-up by KRBP. Patients who were molecularly confirmed as RhD-positive, RhD-negative and DEL and those for whom molecular tests were not performed were excluded. As a result, 58 patients were characterized by molecular methods.

To investigate the prevalence of serological weak D in Koreans, we retrospectively reviewed the electronic medical records of 169,102 patients who visited Seoul National University Bundang Hospital, South Korea, from 2013 to 2018 and who underwent RhD serological testing. For the molecular characterization of the Korean serological weak D phenotype, a total of 105 KRBP reports prepared in response to requests for RhD-variant typing during the same period were analyzed retrospectively. Altogether, 6 RhD-positive, 22 RhD-negative, 11 DEL, and 8 cases for which molecular tests were not performed were excluded. Consequently, 58 cases were molecularly defined as weak D or partial D (Figure 1). This study was approved by the Institutional Review Board of Seoul National University Bundang Hospital (IRB no. B-1910/572-001). The patients’ informed consent was waived due to the retrospective nature of the study.

### 2.2. Serological RhD Tests

**Figure 2:**
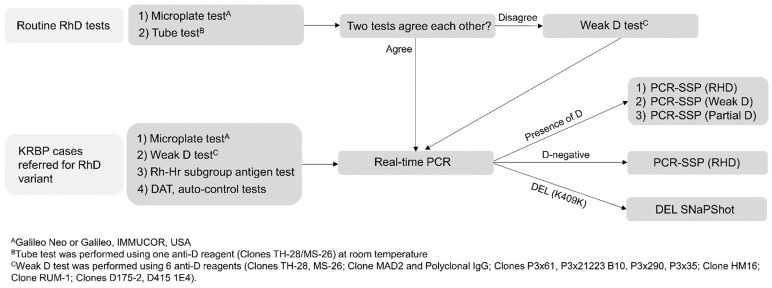
In-house protocol of the RhD variant work-up. In routine RhD tests, microplate tests by automated instrument and tube tests are used at room temperature using a blended IgM/IgG monoclonal anti-D reagent. If two test results revealed serological weak D, RhD genotyping by real-time PCR and PCR- SSP was performed. If the two results disagreed, a weak D test with six kinds of anti-D reagents was carried out, followed by RhD genotyping. Meanwhile, for KRBP cases referred for RhD variant typing, microplate tests, weak D test using six anti-D reagents, RH-Hr subgroup antigen tests, direct antiglobulin tests (DATs), and auto-control tests were conducted. Then, RhD genotyping was performed.

The in-house protocol for RhD-variant work-up is presented in Figure 2. An automated microplate instrument (Galileo Neo or Galileo; Immucor Inc., Norcross, GA, USA) was used for RhD typing in accordance with the manufacturer’s instructions. For routine RhD tube tests, a blended IgM/IgG monoclonal anti-D reagent (Clones TH-28/MS-26; Shinyang Diagnostics, Siheung, Korea) was used initially. Weak D was serologically determined by indirect anti-human globulin tests (IATs) using six anti-D antisera (Table 1) (hereinafter referred to as the Weak D test). Weak D tests were carried out via two methods: tube tests and column agglutination tests (CATs). When agglutination was observed in a tube test or CAT, the result was reported as serological weak D.

For tube tests, a drop of 2–5% RBC suspension and a drop of anti-D antiserum were mixed in a test tube. Bovine albumin 22% solution (Lorne Laboratories, Lower Earley, Berkshire, UK) was used as the negative control. The solution was centrifuged at 3000 rpm for 15 s and the RT phase was interpreted within 2–3 min. Next, the card was incubated at 37 °C for 30–60 min. Following centrifugation at 3000 rpm for 15 s, RBCs with or without bound anti-D antibodies were washed with normal saline three or four times. Next, a drop of AHG (AHG Maestria IgG + C3d; Diagast Laboratoires, Lille, France) was added, and the mixture was centrifuged at 3000 rpm for 15 s. Agglutination was visually inspected and graded according to criteria described by Marsh [14].

For the CAT, two commercial kits were used. A total of 50 μL of 0.85% RBC suspension in low-ionic-strength saline (LISS) was mixed with 25 μL of anti-D antiserum on an LISS/Coombs card (anti-IgG+C3d; Bio-Rad, CA, USA), and the card was incubated at 37 °C for 13 min. The card was then centrifuged at 3000 rpm for 10 s, and agglutination was assessed.

From 2013 to 2014, two ID-cards for partial D identification (ID-Partial D Typing Set, cell lines LHM76/55, LHM77/64, LHM70/45, LHM59/19, LHM169/80, and LDM1; Bio-Rad; and Extended Partial RhD Typing Set, cell lines LHM76/58, LHM76/59, LHM174/102, LHM50/2B, LHM169/81, ESD1, LHM76/55, LHM77/64, LHM70/45, LHM59/19, LHM169/80, and LHM57/17; Bio-Rad) were used according to the manufacturer’s recommendations.

### 2.3. Rh-Hr Subgroup Antigen Tests

Using 25 μL of packed RBCs and 500 μL of normal saline, a 5% RBC suspension was generated. In test tubes, 50 μL of RBC suspension and 50 μL of anti-C, anti-E, anti-c, and anti-e sera (Diagast) were mixed. The tubes were centrifuged at 3000 rpm for 15 s. The agglutination grade was visually assessed and a grade of at least ± (very small aggregates) was reported as positive.

### 2.4. Molecular RhD Tests

#### 2.4.1. Real-Time PCR

In-house real-time PCR was performed to confirm the presence of RhD antigens according to previously published protocols with slight modifications [15]. Briefly, genomic DNA was extracted from whole blood using QIAmps DNA Blood Mini Kit (Qiagen, Hilden, Germany). The targeted regions were *RHD*(K409K), an allelic variant most commonly found in Eastern Asians with DEL, and the 3′-untranslated region (3′-UTR) of RHD exon 10. The β-globin gene was used as an internal control. RhD-positive, RhD-negative, and *RHD*(K409K) samples were used as positive controls to check the quality of the tests. A total of six primers and four probes was used (Supplementary Table S1). PCR was performed on a LightCycler 2.0 (Roche, Basel, Switzerland). For *RHD*(K409K), a 20.0 μL PCR mixture containing 12.4 μL of dextrose water (DW), 2.4 μL of 25 mM MgCl2, 2.0 μL of 10× Master Mix (MMX) (LC FastStart DNA Master HybProbe; Roche), 0.3 μL of each primer, 0.3 μL of each probe, and 2.0 μL of DNA was used. The thermal-cycling conditions were: one cycle of 95 °C for 10 min, 35 cycles of 95 °C for 5 s, 56 °C for 10 s, and 72 °C for 10 s. The melting conditions were: one cycle of 95 °C for 30 s, 40 °C for 4 min, and an increase to 80 °C at a ramp rate of 0.4 °C per s; and one cycle of 40 °C for 10 s. A total of 20.0 μL PCR mixture for analyzing the 3′-UTR of RHD exon 10 and β-globin consisted of 9.8 μL of DW, 4.0 μL of 5× MMX (Roche), 0.4 μL of each primer, 0.4 μL of probe, and 5.0 μL of DNA. The thermal profile was as follows: one cycle of 94 °C for 10 min, 40 cycles of 94 °C for 10 s, 55 °C for 15 s, and 72 °C for 10 s; and one cycle of 40 °C for 30 s. The real-time PCR result was reported as follows: presence of RhD, D-negative (including RHD*08N.01(ψ) and RHD-CE-D hybrid genes as well as homozygous RHD deletion), or DEL. The presence of RhD was further classified as RhD-positive, weak D, or partial D using PCR-SSP.

#### 2.4.2. PCR-SSP

PCR-SSP was performed to identify the RhD subtype using the BAGene RH-TYPE, Partial D-TYPE and/or Weak D-TYPE SSP kits (BAG Health Care GmbH, Lich, Germany). The PCR mixture was composed of 1 μL of 10× PCR buffer, 1 μL of DNA, 0.08 μL of Taq polymerase, and 8 μL of DW. The reaction mixture (final volume 10 μL) was dispensed into reaction tubes containing pre-aliquoted allele and control-specific primers and nucleotides. PCR was performed under the following conditions: denaturation for one cycle at 96 °C for 5 min and five cycles of 96 °C for 10 s and 70 °C for 60 s, followed by 10 cycles of 96 °C for 10 s, 65 °C for 50 s, and 72 °C for 45 s; 15 cycles of 96 °C for 10 s, 61 °C for 50 s, and 72 °C for 45 s; and a final extension for one cycle at 72 °C for 5 min. The amplified products were separated using by electrophoresis using 2% agarose gel with ethidium bromide (0.5 g/mL) at 100 V for 20 min. The results were interpreted according to the worksheet provided by the manufacturer.

## 3. Results

### 3.1. Serology

Of the 169,102 patients, 168,566 (99.68%) patients showed the RhD-positive phenotype and 502 (0.30%) showed the RhD-negative phenotype, while 33 (0.02%) showed the serological weak D phenotype. Consequently, the prevalence of serological weak D in Koreans was estimated to be 0.02%. Among 105 KRBP patients referred for assessment of the RhD variant, 68 had the serological weak D phenotypes, though 10 of them were confirmed to be RhD-positive, RhD-negative, or *RHD* (K409K) via the molecular tests. Six cases were negative for anti-D IgG (Clone HM16; Diagast) using the tube test at RT. All were RhD-positive according to RhD genotyping. Three cases exhibited no reactivity in tube tests but exhibited serological reactivity grade 1+ or 2+ in six CATs. They also tested positive in the DAT, and two tested positive in the auto-control test. Two of the three were confirmed as RhD-negative and one as *RHD* (K409K). Lastly, a case that was graded 2+ in the tube test using a blended IgM/IgG monoclonal anti-D reagent (clone TH-28/MS-26) was revealed to be RhD-negative (Supplementary Table S2).

### 3.2. Molecular Characterization of RhD Variants

Among the 58 KRBP patients with the molecularly defined weak D or partial D phenotypes, nine (15.5%) were classified as weak D type 15, eight (13.8%) were classified as partial DVa or DBS, and four others (6.8%) were classified as partial DVI, partial DVII, weak D type 2, or weak D type 41 or 45.

Of the 58 cases, 11 (19.0%) were defined molecularly as weak D. Weak D type 15 was the most common (n = 9, 81.8%). The following Rh phenotypes were found: ccDEe (n = 4, 44.4%), ccDEE (n = 3, 33.3%), and CcDEe (n = 2, 22.2%). No anti-D antibody was detected. Weak D type 2 was identified in one patient with an Rh phenotype of ccDEe. Auto anti-D formation was noted. Lastly, one patient harbored the CcDee phenotype of weak D type 41 or 45. The case was associated with an atypical real-time PCR melting curve located between the RhD-positive and *RHD* (K409K) curves. The primer sequence of RHK409-F overlaps with the variant site of weak D type 41 (*RHD*, NM_016124: c.1193A>T), which might have resulted in interference during PCR amplification [16].

Of 58 patients, 10 (17.2%) were defined molecularly as partial D. Partial DVa or DBS was the most commonly observed type (n = 8, 80.0%). The Rh phenotypes were CcDEe (n = 3, 30.0%), ccDEe (n = 3, 30.0%), CCDee (n = 1, 10.0%), and CcDee (n = 1, 10.0%). Partial DVI and partial DVII were identified using BAGene Partial D and were associated with the CcDee and CCDee phenotypes, respectively. No anti-D antibody was detected in these 10 molecularly defined partial D cases.

Of 58 cases, 37 (63.8%) were revealed to be either weak D or partial D using molecular methods. The partial D result (DCS (DFW, DHR, DIM, and DNU)) was most common (n = 29, 78.4%) when the BAGene Partial D test was used. Two of those cases harbored auto anti-D. Two patients were found to have *RHD* (K409K) alleles with CcDEe and CCDee phenotypes using BAGene RH. Four (10.8%) unidentifiable cases with CcDee (n = 2), CcDEe (n = 1) and CCDee (n = 1) phenotypes did not produce a sixth band during BAGene Partial D analysis (Table 2).

### 3.3. Correlations between the Serological and Molecular Results

Overall, serological reactivity was inconsistent between and within RhD variant types. Weak D type 2 expressed 1+ or less serological reactivity in tube tests at RT and in immediate spin (IS) tests. Most weak D type 15 cases exhibited no reactivity in six tube tests at RT and in IS tests, but the reactivity increased to 2+ or more in CATs using a blended IgM/IgG monoclonal anti-D reagent (clones TH-28/MS-26). Weak D type 41 or 45 cases exhibited relatively even reactivity to the six anti-D reagents. For partial DVI, reactivity of 2+ or less was observed in tube tests at RT, whereas relatively strong serological reactions were observed for partial DVII (Supplementary Table S3).

### 3.4. Discrepancies between the Serological and Molecular Results

In four cases, there were inconsistent results between serological and molecular tests. A molecularly identified weak D type 15 case was typed as DFR using a Partial D card with six cells (Bio-Rad) but as unidentifiable using a Partial D card with 12 cells (Bio-Rad). Three undistinguishable cases by molecular tests were analyzed as DV or unidentifiable using a Partial D card with six cells (Bio-Rad) but as DV, DIII, or unidentifiable using a Partial D card with 12 cells (Bio-Rad) (Table 3).

## 4. Discussion

In this study, the prevalence of serological weak D in Koreans was estimated to be approximately 0.02%, lower than in Caucasians (0.2–1.0%) [3]. Weak D type 15 was the most common, in accordance with a previous report [7]. However, partial DVa or DBS was the most common partial D type, although the sub-alleles were not identifiable due to methodological limitations. Partial D type VI is reportedly the most common in South Korea based on serological methods [17].

A limited number of RhD variant types could be detected presumably due to a limitation in the PCR-SSP kits using pre-set primers targeting specific regions. Thirty-seven undistinguishable cases might have harbored rare or unknown alleles not detectable using the PCR-SSP kit. However, it was necessary to rule out weak D types 1, 2, and 3, which can be treated as RhD-positive. Thus, in an urgent situation in which molecular RhD typing cannot be performed, it would be safe to release RhD-negative RBCs for Koreans with serological weak D.

Some discordant results between Partial D cards and PCR-SSP were observed (Table 3). The types determined using the Partial D cards were detectable via PCR-SSP. We provided only four cases which were available retrospectively, but more cases would have been identified if further testing was performed. The discrepant results were likely caused by the limitations of the serological techniques, further emphasizing the importance of RhD genotyping.

Our routine molecular RhD-typing protocols have a fundamental limitation. If only samples with serological weak D of 2+ or less reactivity were tested, serologically positive RhD variants that exhibit strong reactivity to anti-D reagents might be missed. RhD variants with the N152T amino-acid substitution, such as DNT, DIII type 4, DIV type 1, and DVI type III, have the same antigen sites per RBC as those found in normal D-positive RBCs [18]. These partial D types would not be detected until allo anti-D is produced to a detectable level. Consequently, the prevalence of serological weak D as calculated based on our data may be an underestimate.

## 5. Conclusions

In conclusion, the prevalence of serological weak D in Koreans was estimated to be approximately 0.02%. The most frequent RhD variant types were weak D type 15 and partial DVa or DBS. This suggests that serological weak D in Koreans should be treated as RhD-negative in most cases. However, molecular confirmation is needed for an individualized transfusion strategy. Our data suggest that ethnic differences exist in the prevalence of serological weak D and incidence of weak D and partial D. Thus, we recommend the establishment of an ethnicity-focused database.

## Figures and Tables

**Table 1:** Anti-D reagents.

Number	Immunoglobulin	Clone	Manufacturer
1	IgM + IgG	TH-28, MS-26	Shinyang Diagnostics, Siheung, Korea
2	IgM + IgG	MAD2, polyclonal IgG	Ortho Clinical Diagnostics, Raritan, New Jersey, USA
3	IgM + IgG	P3x61, P3x21223 B10, P3x290, P3x35	Diagast Laboratoires, Lille, France
4	IgG	HM16	Diagast Laboratoires, Lille, France
5	IgM	RUM-1	Immucor, Norcross, GA, USA
6	IgM + IgG	D175-2, D415 1E4	Immucor, Norcross, GA, USA

**Table 2:** Molecular characteristics of 58 serological weak D patients as elucidated using real-time polymerase chain reaction (PCR) and PCR with sequence-specific primers (PCR-SSP), including information on anti-D antibody formation and the Rh phenotype.

D Variant	ISBT Nomenclature	Patients (n, %)	RhD Genotype	BAGene RH	BAGene Partial D	BAGene Weak D	Anti-D Ab	Phenotype (n)
Weak D type 2	RhD*01W.2	1 (9.1)	Presence of D	D-positive	D-positive or Partial D (DCS (DFW, DHR, DIM, DNU))	RHD*weak D type 2	Auto anti-D	ccDEe (1)
Weak D type 15	RhD*15	5 (45.5)	Presence of D	D-positive	D-positive or Partial D (DCS (DFW, DHR, DIM, DNU))	RHD*weak partial 15	None	CcDEe (2), ccDEe (4), ccDEE (3)
		4 (36.4)	Presence of D	NT	D-positive or Partial D (DCS (DFW, DHR, DIM, DNU))	RHD*weak partial 15	None	
Weak D type 41 or 45	RhD*01W.41 or RhD*01W.45	1 (9.1)	Presence of D	D-positive	RHD (delEx9)	NT	None	CcDee (1)
Total weak D (%)		11 (100.0)						
Partial DVa or DBS	RhD*05 or RhD*13 ^1^	2 (20.0)	Presence of D	D-positive	Partial D (DVa, Va-like, Va-associated, DBS)	RhD-positive or RhD-negative	None	CcDEe (3), ccDEe (3), CCDee (1), CcDee (1)
		3 (30.0)	Presence of D	NT	Partial D (DVa, Va-like, Va-associated, DBS)	NT	None	
		3 (30.0)	NT	NT	Partial D (DVa, Va-like, Va-associated, DBS)	NT	None	
Partial DVI	RhD*06 ^1^	1 (10.0)	Presence of D	DVI	DVI type 3	RhD-positive or RhD-negative	None	CcDee (1)
Partial DVII	RhD*07 ^1^	1 (10.0)	Presence of D	D-positive	DVII	RhD-positive or RhD-negative	None	CCDee (1)
Total partial D (%)		10 (100.0)						
Weak D or partial D	N/A	14 (24.1)	Presence of D	D-positive	D-positive or Partial D (DCS (DFW, DHR, DIM, DNU))	RhD-positive or RhD-negative	Auto anti-D	CcDEe (2), ccDEe (2), CcDee (4), CCDee (4), ccDEE (2)
		5 (8.6)	Presence of D	NT	D-positive or Partial D (DCS (DFW, DHR, DIM, DNU))	RhD-positive or RhD-negative	None	CcDEe (1), ccDEe (2), CcDee (1), ccDEE (1)
		3 (5.2)	Presence of D	NT	D-positive or Partial D (DCS (DFW, DHR, DIM, DNU))	NT	Auto anti-D	CcDEe (1), CCDee (1), ccDEE (1)
		4 (6.9)	NT	NT	D-positive or Partial D (DCS (DFW, DHR, DIM, DNU))	RhD-positive or RhD-negative	None	ccDEe (3), CCDee (1)
		2 (3.4)	NT	NT	D-positive or Partial D (DCS (DFW, DHR, DIM, DNU))	NT	None	CcDee (1), CCDee (1)
		1 (1.7)	Presence of D	D-positive (*RHD*/*RHD*(K409K))	D-positive or Partial D (DCS (DFW, DHR, DIM, DNU))	NT	None	CcDEe (1)
		1 (1.7)	Presence of D	D-positive (*RHD*/*RHD*(K409K))	NT	RhD-positive or RhD-negative	None	CCDee (1)
		2 (3.4)	Presence of D	NT	Unidentifiable ^2^	RhD-positive or RhD-negative	None	CcDee (2)
		1 (1.7)	NT	NT	Unidentifiable ^2^	NT	None	CCDee (1)
		1 (1.7)	NT	NT	Unidentifiable ^2^	RhD-positive or RhD-negative	None	CCDee (1)
		1 (1.7)	Presence of D	NT	NT	RhD-positive or RhD-negative	None	CcDEE (1)
Total weak D or partial D (%)		37 (100.0)						
Total D variants		58						

Ab: antibody, ISBT: International Society of Blood Transfusion, N/A: not applicable, NT: not tested. ^1^ Unidentified suballeles. ^2^ Four unidentifiable cases did not produce a sixth band in the BAGene Partial D test.

**Table 3:** Discrepancies in the results of tests using Partial D cards and PCR-SSP.

Molecular Identification	Partial D Card (6 Cells)	Partial D Card (12 Cells)
Weak D type 15	DFR	Unidentifiable
Weak D or partial D	DV	DV
Weak D or partial D	Unidentifiable	DIII
Weak D or partial D	DV	Unidentifiable

## Supplementary Materials

The following are available online at https://www.mdpi.com/article/10.3390/diagnostics11060920/s1, Table S1: Primers and probes used for real-time PCR, Table S2: False serological weak D results produced by tube tests using six anti-D reagents at room temperature, column agglutination tests, direct antiglobulin tests, auto-control tests, and RhD genotyping. Table S3: Serological reactivity exhibited by D variants in tube tests using six anti-D reagents at room temperature, column agglutination tests, and immediate spin tests.
