# Supplementary material for: Molecular Characteristics of the Serological Weak D Phenotype in Koreans
Source: Diagnostics (Basel). 2021 May 21;11(6):920. doi: 10.3390/diagnostics11060920 (PMC8223775; doi:10.3390/diagnostics11060920)
Supplement: Supplementary file 1 [file diagnostics-11-00920-s001.zip › diagnostics-1224033-supplementary.pdf]

Supplementary Materials

Table S1. Primers and probes used for real-time PCR

Primer or probe	Nucleotide sequence
RHK409-F	5'-AAAATATGGAAAGCACCTCATGA-3'
RHK409-R	5'-ATGGATTTGTTTCTCCTCTAGTT-3'
RHK409-D	LC Red 640-ATCTTACCTTCCAGAAAACTTGGTCATCAA-Phosphate
RHK409-A	CATGCACTCAAAATCTATCACGTTAATAGGTGAA-Fluorescein
RHZ10-F	5'-CCTCTCACTGTTGCCTGCATT-3'
RHZ10-R	5'-AGTGCCTGCGCGAACATT-3'
RHZ10-P	5'-FAM-TACGTGAGAAACGCTCATGACAGCAAAGTCT-TAMRA-3'
BGB-F	5'-CTGCACCTGACTCCTGAGGAGA-3'
BGB-R	5'-CCTTGATACCAACCTGCCCAG-3'
BGB-P	5'-FAM-AAGGTGAACGTGGATGAAGTTGGTGG-p3'

Table S2. False serological weak D results produced by tube tests using six anti-D reagents at room temperature, column agglutination tests, direct antiglobulin tests, auto-control tests, and RhD genotyping

	T-1	T-2	T-3	T-4	T-5	T-6	C-1	C-2	C-3	C-4	C-5	C-6	DAT	Auto	RhD genotyping
Immunoglobulin	IgM+IgG	IgM+IgG	IgM+IgG	IgG	IgM	IgM+IgG	IgM+IgG	IgM+IgG	IgM+IgG	IgG	IgM	IgM+IgG			
Clone	TH-28, MS-26	MAD2, polyclonal IgG	P3x61, P3x21223 B10, P3x290, P3x35	HM16	RUM-1	D175-2, D415 1E4	TH-28, MS-26	MAD2, polyclonal IgG	P3x61, P3x21223 B10, P3x290, P3x35	HM16	RUM-1	D175-2, D415 1E4			
Group 1	4+	3+	4+	−	4+	4+	4+	3+	4+	3+	4+	4+	−	−	RhD-positive
	4+	3+	4+	−	4+	4+	4+	3+	4+	3+	4+	4+	−	−	RhD-positive
	4+	4+	4+	−	4+	4+	4+	3+	4+	4+	4+	4+	−	−	RhD-positive
	4+	4+	4+	−	4+	4+	4+	3+	4+	3+	4+	4+	−	−	RhD-positive
	4+	4+	4+	−	4+	4+	4+	4+	4+	4+	4+	4+	−	−	RhD-positive
	4+	4+	4+	−	4+	4+	4+	3+	4+	3+	4+	4+	−	−	RhD-positive
Group 2	−	−	−	−	−	−	1+	1+	1+	1+	1+	1+	+	−	RhD-negative
	−	−	−	−	−	−	1+	1+	1+	1+	1+	1+	+	+	RhD-negative
	−	−	−	−	−	−	2+	2+	2+	2+	2+	2+	+	+	RHD(K409K)
Group 3	2+	−	−	−	−	−	−	−	−	−	−	−	−	−	RhD-negative

Auto: auto-control test, C:column agglutination test, DAT: direct antiglobulin test, T: tube test.

Table S3. Serological reactivity exhibited by D variants in tube tests using six anti-D reagents at room temperature, column agglutination tests, and immediate spin tests.

	T-1	T-2	T-3	T-4	T-5	T-6	C-1	C-2	C-3	C-4	C-5	C-6	IS-D	IS-D′
Immunoglobulin	IgM+IgG	IgM+IgG	IgM+IgG	IgG	IgM	IgM+IgG	IgM+IgG	IgM+IgG	IgM+IgG	IgG	IgM	IgM+IgG	IgM	IgM+IgG
Clone	TH-28, MS-26	MAD2, polyclonal IgG	P3x61, P3x21223 B10, P3x290, P3x35	HM16	RUM-1	D175-2, D415 1E4	TH-28, MS-26	MAD2, polyclonal IgG	P3x61, P3x21223 B10, P3x290, P3x35	HM16	RUM-1	D175-2, D415 1E4	P3x61	P3x61, P3x21223 B10, P3x290, P3x35
Weak D type 2	±	–	–	1+	1+	1+	3+	2+	2+	3+	2+	2+	1+	1+
Weak D type 15	–	–	–	–	–	–	3+	2+	1+	–	–	2+	–	–
	–	–	–	–	–	–	3+	2+	2+	–	–	2+	–	–
	±	–	–	–	–	–	2+	±	–	–	–	±	–	–
	–	–	–	–	–	–	2+	1+	±	–	–	±	–	–
	–	–	–	–	–	–	2+	±	–	–	–	±	–	–
	–	–	–	–	–	–	3+	3+	3+	1+	–	3+	–	–
	–	–	–	–	–	–	1+	1+	1+	–	–	1+	–	–
	–	–	–	–	–	–	2+	1+	1+	–	–	1+	–	–
	–	–	–	–	–	–	2+	1+	2+	NT	–	1+	–	–
Weak D type 41 or 45	1+	1+	1+	NT	2+	1+	3+	2+	3+	NT	3+	3+	2+	2+
Partial DVa or DBS	3+	1+	4+	–	4+	4+	4+	3+	4+	3+	4+	4+	4+	3+
	NT	NT	NT	NT	NT	NT	3+	2+	3+	–	–	3+	–	±
	–	–	1+	NT	–	–	4+	3+	3+	–	–	4+	–	2+
	2+	–	2+	–	2+	2+	4+	3+	3+	3+	3+	3+	3+	3+
	–	NT	–	–	–	–	4+	NT	4+	–	–	3+	–	3+
	–	–	3+	–	–	–	4+	3+	4+	–	–	3+	–	3+
	–	–	–	–	–	–	3+	1+	2+	±	±	2+	–	–
	–	–	–	–	–	–	3+	2+	3+	–	–	3+	–	1+
Partial DVI	–	–	2+	–	–	–	4+	2+	3+	–	–	4+	–	4+
Partial DVII	4+	3+	4+	–	4+	4+	4+	3+	4+	3+	4+	4+	4+	4+
Weak D or partial D	–	–	–	–	±	±	3+	3+	2+	3+	2+	2+	–	2+
	–	–	–	–	–	–	2+	2+	±	2+	–	±	–	–

±	1+	1+	−	1+	1+	4+	3+	3+	4+	4+	4+	3+	3+
±	−	−	−	±	±	3+	3+	2+	1+	3+	2+	−	−
−	−	−	−	±	±	4+	3+	2+	2+	3+	4+	−	−
−	−	−	−	−	−	2+	1+	±	−	−	1+	−	−
−	−	−	−	−	−	1+	1+	−	1+	−	±	−	−
±	−	±	±	2+	2+	4+	3+	2+	3+	3+	3+	2+	2+
±	±	±	−	±	±	2+(mf)	2+(mf)	2+(mf)	2+(mf)	2+(mf)	2+(mf)	4+	4+
−	−	−	NT	2+	2+	4+	3+	3+	NT	3+	4+	±	±
2+	2+	2+	−	2+	2+	4+	4+	4+	4+	4+	4+	4+	4+
−	−	−	−	±	±	2+	2+	2+	3+	3+	2+	2+	1+
±	−	±	−	1+	1+	3+	2+	2+	3+	3+	2+	2+	2+
−	−	−	−	±	±	4+	2+	3+	2+	2+	3+	−	−
−	NT	−	−	−	−	2+	NT	1+	NT	1+	2+	NT	NT
3+	2+	3+	−	4+	4+	3+	2+	3+	4+	4+	4+	3+	4+
3+	2+	3+	−	4+	4+	3+	2+	3+	3+	4+	4+	3+	3+
3+	−	4+	NT	3+	4+	3+	2+	3+	NT	4+	4+	3+	4+
1+	±	2+	−	2+	2+	4+(mf)	2+	4+(mf)	3+	4+(mf)	4+(mf)	3+(mf)	3+(mf)
±	±	±	−	2+	1+	3+	2+	3+	3+	3+	3+	±	±
−	−	−	−	1+	1+	3+	2+	3+	3+	3+	4+	±	−
±	−	−	−	±	−	3+	2+	3+	3+	3+	3+	2+	2+
−	−	3+	−	−	−	3+	2+	3+	−	−	3+	−	3+
−	−	−	−	−	−	1+	1+	±	2+	−	1+	−	−
4+	3+	4+	−	4+	4+	4+	3+	4+	4+	4+	4+	3+	3+
1+	1+	2+	NT	3+	3+	4+	3+	3+	NT	4+	4+	3+	3+
−	−	−	−	−	−	3+	2+	3+	2+	2+	3+	−	−
−	−	−	−	−	−	3+	2+	3+	2+	2+	3+	±	−
−	−	−	−	3+	1+	3+	2+	3+	2+	2+	3+	−	−
−	−	−	−	−	−	±	1+	±	1+	−	±w	−	−
−	−	−	−	−	−	±	1+	−	2+	−	−	−	−
−	−	−	−	−	±	2+	2+	2+	2+	1+	2+	−	−
1+	1+	3+	−	1+	2+	4+	2+	3+	−	−	4+	3+	3+
1+	1+	2+	−	2+	3+	3+(mf)	2+(mf)	2+(mf)	2+(mf)	2+(mf)	2+(mf)	2+(mf)	2+(mf)
4+	3+	3+	3+	3+	4+	4+	4+	4+	4+	4+	4+	NT	NT
3+	3+	3+	−	4+	4+	4+	4+	4+	3+	4+	4+	4+(mf)	4+(mf)
3+	2+	2+	−	4+	4+	4+	4+	4+	4+	4+	4+	4+	4+

C: column agglutination test, IS: immediate spin, mf: mixed field, NT: not tested, T: tube test, w: weak.
